# Growth and characterization of gold catalyzed SiGe nanowires and alternative metal-catalyzed Si nanowires

**DOI:** 10.1186/1556-276X-6-187

**Published:** 2011-03-01

**Authors:** Alexis Potié, Thierry Baron, Florian Dhalluin, Guillaume Rosaz, Bassem Salem, Laurence Latu-Romain, Martin Kogelschatz, Pascal Gentile, Fabrice Oehler, Laurent Montès, Jens Kreisel, Hervé Roussel

**Affiliations:** 1 LTM/CNRS-CEA-LETI, 17, rue des martyrs, 38054 Grenoble, France; 2 CEA/INAC/SiNaPS, 17, rue des martyrs, 38054 Grenoble, France; 3 IMEP-LAHC, Grenoble Institute of Technology, MINATEC, BP 257, 3 parvis Louis NEEL 38016 Grenoble, France; 4 LMGP, CNRS, Grenoble Institue of Technology, 3 parvis Louis Néel, 38016 Grenoble, France

## Abstract

The growth of semiconductor (SC) nanowires (NW) by CVD using Au-catalyzed VLS process has been widely studied over the past few years. Among others SC, it is possible to grow pure Si or SiGe NW thanks to these techniques. Nevertheless, Au could deteriorate the electric properties of SC and the use of other metal catalysts will be mandatory if NW are to be designed for innovating electronic. First, this article's focus will be on SiGe NW's growth using Au catalyst. The authors managed to grow SiGe NW between 350 and 400°C. Ge concentration (*x*) in Si_1-__*x*_Ge_*x *_NW has been successfully varied by modifying the gas flow ratio: *R *= GeH_4_/(SiH_4 _+ GeH_4_). Characterization (by Raman spectroscopy and XRD) revealed concentrations varying from 0.2 to 0.46 on NW grown at 375°C, with *R *varying from 0.05 to 0.15. Second, the results of Si NW growths by CVD using alternatives catalysts such as platinum-, palladium- and nickel-silicides are presented. This study, carried out on a LPCVD furnace, aimed at defining Si NW growth conditions when using such catalysts. Since the growth temperatures investigated are lower than the eutectic temperatures of these Si-metal alloys, VSS growth is expected and observed. Different temperatures and HCl flow rates have been tested with the aim of minimizing 2D growth which induces an important tapering of the NW. Finally, mechanical characterization of single NW has been carried out using an AFM method developed at the LTM. It consists in measuring the deflection of an AFM tip while performing approach-retract curves at various positions along the length of a cantilevered NW. This approach allows the measurement of as-grown single NW's Young modulus and spring constant, and alleviates uncertainties inherent in single point measurement.

## Introduction

Owing to their novel and promising potential applications for upcoming technologies, semiconductor (SC) nanowires (NW) have been the object of an increasing interest during the past few years. Indeed, numerous publications show the diversity of applications these nanostructures are destined to: electronic devices [1-3], optoelectronics and photonics [4-6], sensors [7,8], solar cells [9-11], etc. The existing NW synthesis methods are numerous, and each one has its own advantages and drawbacks. Top-down approach uses well-mastered lithography and etching techniques to build nanostructures from an existing substrate. The technologies used allow the design of advanced devices [12], but this approach is limited by its advantages: the limits of lithography and etching techniques and the use of an existing crystalline material which makes it difficult to vary composition, specifically for 3D and back-end integration. Bottom-up approach, which will be the focus of this study, allows the growth of a crystalline nanostructure on any substrate at low temperatures. The material is supplied by external means and can be varied to modify the nanostructure's composition, and the dimension of the object can be very small. However, the localization of the nanostructures and the CMOS compatibility of these techniques constitute serious challenges. One of the most-cited methods is the so-called vapour-liquid-solid growth first reported by Wagner and Ellis in 1964 [13]. This method is based on a catalyzed deposition of the SC precursor on a liquid metal droplet, which allows the growth rate to be orders of magnitude higher in one direction than in the others. In the case of Si and Ge SCs, gold is often used as an efficient catalyst. The physical properties of Si and Ge make it possible to synthesize a wide range of composition alloys as well as a variety of structures using Si, Ge, and SiGe alloy. The SiGe alloy allows band gap engineering and improved carrier mobility with applications in high-speed electronics or optoelectronics [14,15] because of the CMOS compatibility of the alloy. Furthermore, it is possible to synthesize SiGe NW to combine the properties of this alloy to the numerous promising 1D applications for 3D electronics. However, it is mandatory to control the alloy composition of such structures. Synthesis by chemical vapor deposition (CVD) has already been demonstrated by different groups in the past [16-19]. In this study, SiGe NW synthesis down to 350°C with a Ge concentration ranging from 0 to 50% is reported.

However, it is important to keep in mind that the catalyst material is expected to be more or less incorporated into the NW during growth. Gold is known to create deep traps in the band gap decreasing the carrier mobility and lifetime in Si and Ge, and be responsible for serious problems of contamination for the CMOS technology. Si NW growths using alternative metal catalysts have already been reported previously with Pt [20], Al [21], Cu [22], Ti [23], Pd [24], Mn [25], and Fe [26]. The temperatures needed are much higher with those metals than for gold because of the physical properties of the alloy catalyst particles. The eutectic temperatures of alloy involving such metals are much higher than for gold. In most of the cases, the catalyst island remains solid during the growth (VSS process) which also implies high growth temperatures. Uncatalyzed growth rate dramatically increases with temperature inducing an important tapering of the NW. In this study, the growth of Si NW catalyzed by PtSi, NiSi, and Pd_2_Si is reported. The use of gaseous HCl as a means to prevent Si deposition on the sidewalls of the NW responsible for the tapering effect is introduced. Finally, as NW are also destined to be components for NEMS [27], AFM-based mechanical characterization has also been carried out on Si and GaN NW for comparison.

### SiGe NW growth

First, the growth of SiGe NW using gold as catalyst is reported. Gold is particularly suitable for SiGe growth because the proportions and temperatures of the eutectic metal/SC alloy needed are approximately the same for Au/Si and Au/Ge (80 and 70% Au, 360°C) [28]. With this eutectic temperature being much lower than those of the silicides, the NW are synthesized via the VLS process: the liquid metal/SC alloy droplets on the substrate act as preferred sites for the adsorption and decomposition of the gaseous precursor. When the alloy droplets are saturated with the SC atoms, they precipitate at the liquid/solid interface to form the NW. NW's structural properties have been characterized by scanning electron microscopy (SEM), transmission electron microscopy (TEM), and X-ray diffraction (XRD).

The samples of SiGe NW described in this study were grown in a reduced-pressure CVD system on Si (111) substrates. A 2-nm Au layer is deposited by evaporation after a proper cleaning step. The substrate is then loaded into the deposition chamber and annealed at 650°C for several minutes in order to dewet the gold layer and form the Au/Si droplets. Then, the temperature is cooled down to the deposition temperature. In this study, the reactor temperature is varied from 325 to 450°C. The total pressure is fixed at 4.5 Torr, and the flow of the Hydrogen carrier gas (H_2_) is maintained at 1900 sccm. Si and Ge are provided, respectively, by pure silane (SiH_4_) and germane (GeH_4 _5% in H_2_). The NW's morphology, dimensions, and density are characterized by SEM. Their crystalline quality and orientation are determined by means of TEM images. The composition *x *of the Si_1-__*x*_Ge_*x *_alloy NW is determined using XRD applying the Vegard's law and Raman spectroscopy. To determine *x *according to this technique, the shift of the Si-Si peak is used. Indeed, an SiGe Raman spectrum displays different peaks corresponding to the Ge-Ge, Ge-Si, or Si-Si bonds. In this case, the Si-Si peak from the SiGe NW is shifted to the left of the Si-Si peak from the substrate. The shift between those two peaks allows us to determine the percentage of Ge in the alloy [29].

First, the composition of the SiGe NW has been studied as a function of the temperature and of the gas ratio: *R *= *P*_GeH4_/(*P*_SiH4 _+ *P*_GeH4_), where *P*_*X *_is the partial pressure of the precursor *X*. The germane partial pressure is fixed at 10 mTorr, and the silane partial pressure is varied from 55 to 194 mTorr (*R *varies from 0.15 to 0.048).

**Figure 1:**
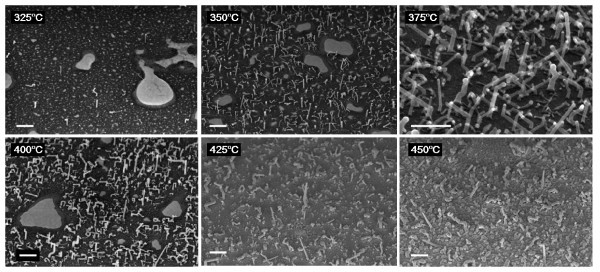
**SEM images of Au-catalyzed SiGe NW grown for 40 min at various temperatures with *R *= 0.15**. Straight NW growth with a good density occurs between 350 and 400°C. For higher temperatures, 2D growth becomes too important thus decreasing NW density. At *T *= 325°C, the temperature seems too low to get a satisfying density. The scale bars are 400 nm.

The influence of temperature has been studied for a constant *R *= 0.15 (*P*_SiH4 _= 55 mTorr). Figure 1 shows the SEM images of the NW grown for 40 min at temperatures varying from 325 to 450°C. As one can see, at high temperatures, the uncatalyzed growth becomes too important and inhibits the growth of NW above 400°C, whereas temperatures below 350°C lead to a very slow growth (poor density and small length). As the process window for SiGe NW seems to be shallow, the growth temperature for the rest of the study will be restricted between 350 and 400°C.

**Figure 2:**
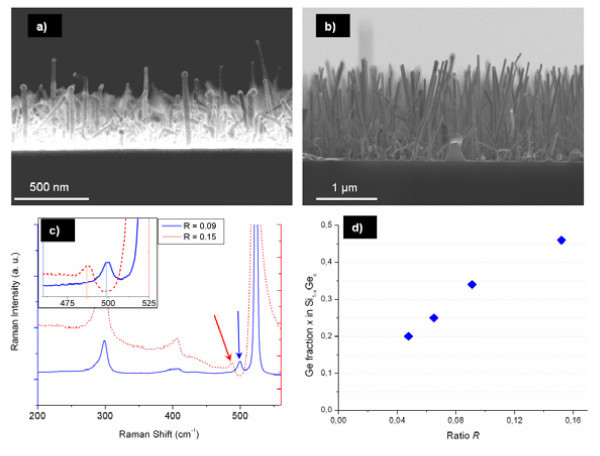
**SEM images, Raman spectra and Ge fraction of SiGe NW**. SEM images of SiGe NW grown during 40 min at 375°C with **(a) ***R *= 0.15 and **(b) ***R *= 0.09. **(c) **Raman spectra collected from samples **(a,b)**. Arrows are pointing at the Si-Si peaks in SiGe used for calculating the Ge fraction. The inset highlights the peaks' shift between two different compositions (Raman shift = 488 cm^-1 ^for *R *= 0.15 and 499 cm^-1 ^for *R *= 0.09). **(d) **Representation of the Ge composition of the SiGe NW as a function of *R*.

To change the Ge composition of the NW, the gas ratio *R *is varied at a constant temperature of 375°C. Figure 2 shows NW grown with *R *= 0.15 and *R *= 0.09 and their respective Raman spectra. It was observed that the NW diameters vary from 20 to 60 nm, whatever be the growth conditions. The growth speed increases linearly from 15 to 75 nm min^-1 ^when *R *decreases from 0.15 to 0.048. This increase can be imputed to the increase of the SiH_4 _partial pressure and thus of the silane deposition rate. DRX and Raman measurements revealed that the Ge concentration (*x*) of the Si_1-x_Ge_*x *_NW has been successfully varied from 0.2 to 0.46 with *R *varying from 0.048 to 0.15, respectively (Figure 2d).

Finally, the Ge concentration as a function of the temperature (350, 375, 400°C) has been studied for *R *= 0.09 and 0.15 (*P*_SiH4 _= 55 and 100 mTorr). The alloy composition shows little variation according to growth temperature for *R *= 0.09. For *R *= 0.15, it reaches 0.52 at 350°C, compared to 0.46 at 375 and 400°C. It is known that activation energy for the decomposition is larger for silane than for germane [16]. The increase in Ge composition has already been observed [30], which could be explained by a lessened decomposition of the silane at low temperature whereas germane decomposition is not affected.

### Silicide catalyst for Si NW growth

In the next section, it will be shown that silicon NW can be grown by CVD using fully CMOS-compatible catalysts: PtSi, Pd_2_Si, and NiSi. These silicides are chosen because they are already present in the CMOS fabrication processes. Silicon NW have been grown on Si(100) by CVD using SiH_4 _as the silicon gas precursor, and H_2 _as the carrier gas. The growth temperature varied between 500 and 800°C and growths were carried out with or without gaseous hydrochloric acid (HCl). The total pressure is maintained at 15 Torr unless otherwise stated.

### PtSi catalyst

PtSi islands, used as the catalyst [20], have been synthesized according to the now described method. Before NW growth, the (100)-Si substrate has been covered with a thin (few nanometres) Pt layer obtained by physical vapor deposition. PtSi was formed by thermal annealing under inert atmosphere at high temperature, and unreacted Pt was removed chemically after the annealing step. The sample was then transferred from the silicide furnace into the CVD reactor after an HF-last cleaning step. Annealing is then adjusted to obtain particles <100 nm. For instance, mean size is 45 nm diameter by 5 nm height. XRD measurements after annealing show that the islands are crystalline PtSi with two main growth directions [101] and [200].

**Figure 3:**
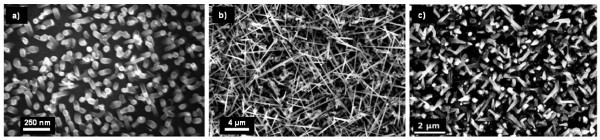
**SEM images of PtSi-catalyzed Si NW grown for 30 min at various temperatures**: **(a) **500°C, **(b) **700°C, **(c) **800°C. P_SiH4_ is held constant at 60 mTorr. The NW grown at 700 and 800°C show a tapered shape, whereas the diameter of the NW grown at 500°C is constant (45 nm).

After island's formation, SiH_4 _in H_2 _is introduced into the deposition chamber and the growth is studied as a function of the temperature. As one can see in Figure 3, the NW grown at low temperature have a constant diameter along their length whereas growth at higher temperatures results in highly tapered NW. This effect could be explained by uncatalyzed growth on the sidewalls of the NW. The vertical growth rate was estimated at 190 nm min^-1^, and the lateral growth rate at 6 nm min^-1 ^(*T *= 700°C; silane partial pressure *P*_SiH4 _= 60 mTorr). Another explanation would be the incorporation of the catalyst into the NW resulting in a diminution of its diameter during growth. This phenomenon might not be predominant because the diameter of the NW tip is the same as the initial catalyst island (45 nm).

**Figure 4:**
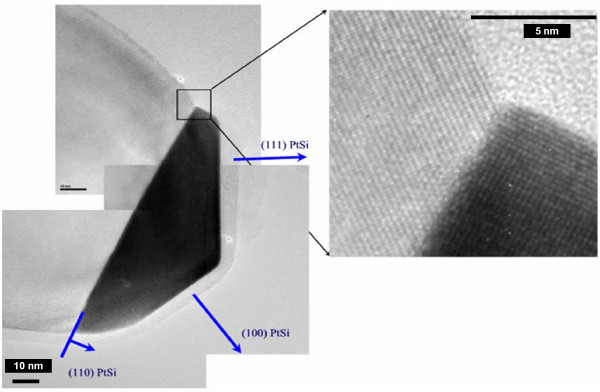
**TEM image of a silicon NW (*T *= 800°C, *P***_**SiH4 **_**= 60 mTorr, *P***_**HCl **_**= 60 mTorr, 30 min) with PtSi catalyst at the top**. The image shows a clearly faceted catalyst, suggesting that it remains solid during growth.

Since the temperatures investigated are less than the PtSi/Si eutectic temperature (979°C), NW are expected to grow via the vapour-solid-solid (VSS) mechanism. During the VSS growth, the catalyst remains solid at the top of the NW and enhances the adsorption and decomposition of the precursor. Figure 4 shows a TEM image of the PtSi catalyst at the top of a Si NW, which supports the previous hypothesis. Indeed, the catalyst particle is clearly crystalline. Unlike Au, PtSi does not form a spherical cap on the top of the NW. It remains strongly faceted or flat suggesting that catalyst does not melt - otherwise, surface tension forces would favor a spherical profile.

It is possible to grow silicon NW with PtSi between 500 and 800°C but uncatalyzed deposition rate at such temperatures becomes a serious issue responsible for the growth of a thick layer and for an important tapering of the NW.

**Figure 5:**
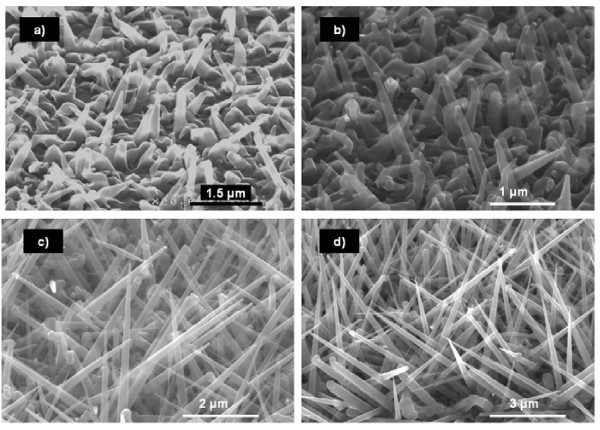
**SEM images of PtSi-catalyzed Si NW grown at 800°C for 10 min with different HCl partial pressures**: **(a) **no HCl, **(b) ***P*_HCl _= 40, **(c) ***P*_HCl _= 100, **(d) ***P*_HCl _= 160 mtorr. Mean aperture angles **(A) **have been measured at the tip of the NW for each sample: **(a) ***A *= 14.4°, **(b) ***A *= 6.6°, **(c) ***A *= 3.4°, and **(d) ***A *= 2.7°. The aperture angle decreases when *P*_HCl _increases, which implies that the tapering effect is considerably reduced using gaseous HCl.

To improve the growth selectivity, HCl gas is introduced into the deposition chamber along with SiH_4_. Figure 5 shows four NW samples grown without HCl and with three different HCl partial pressures (*P*_HCl _= 40, 100, and 160 mTorr). The NW are more or less cone shaped, and the mean aperture angle (formed by the sidewalls of the NW) has been measured on each sample. The mean aperture angle decreases from 14.4° without HCl to 2.7° with *P*_HCl _= 160 mTorr. The aperture angle is a measurement of the tapering of the NW. One can see that the tapering effect is reduced when *P*_HCl _increases, which is most probably due to a Cl surface coverage that inhibits the Si deposition on the sidewalls [31].

### NiSi catalyst

NiSi islands have also been used to catalyze the growth of Si NW. The islands formation method and the experimental protocol are the same as for PtSi. XRD measurements after annealing of the NiSi thin layer show that the islands are orthorhombic NiSi.

**Figure 6:**
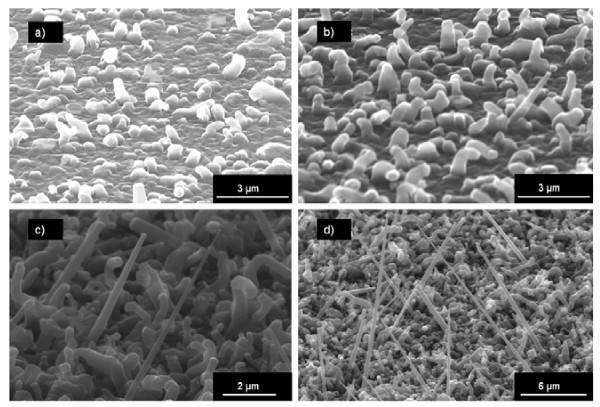
**SEM images of NiSi-catalyzed NW grown at 800°C for 10 min with different HCl partial pressures**: **(a) **no HCl, **(b) ***P*_HCl _= 40, **(c) **P_HCl _= 100, **(d) ***P*_HCl _= 160 mTorr. The lengths of straight NW are 4 μm for **(c) **and 8 μm for **(d)**.

As for PtSi-catalyzed NW, the influence of *P*_HCl _and temperature on the NiSi-catalyzed NW morphology has been studied. First, *P*_HCl _has been varied from 0 to 160 mTorr, *P*_SiH4_, with temperature and deposition time being held constant. Figure 6 shows SEM images of the NW. As one can see, the length and density of the NW increase with *P*_HCl_. From *P*_HCl _= 100 mTorr, straight NW can be observed.

Second, temperature has been varied from 500 to 800°C, at constant HCl and silane partial pressures (respectively, 160 and 100 mTorr) and fixed deposition time (results not shown). It is observed that NW growth occurs from 600°C, and the length and density of the NW increase with the temperature. Straight NW can be observed from 700°C.

### Pd_2_Si catalyst

Finally, the growth of Si NW using Pd_*x*_Si_*y *_island catalysts is reported. The catalyst islands have been formed in the same fashion as presented above, and the experimental protocol remains identical.

**Figure 7:**
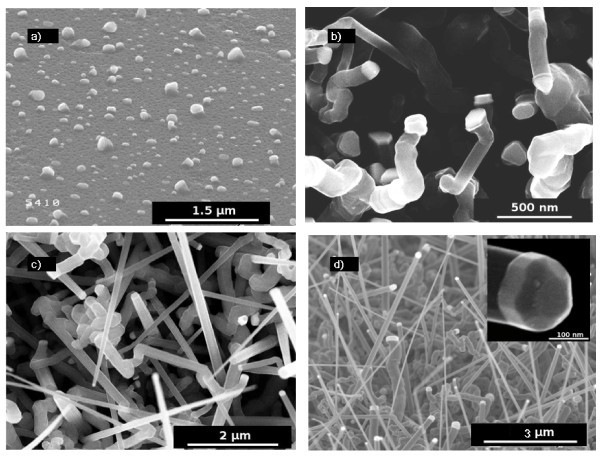
**SEM images of PdSi-catalyzed NW**. (a-c) are grown at *P*_tot _= 15 Torr, and a ratio P_HCl_/P_SiH4 _= 3.3 (200 mTorr/60 mTorr) for 10 min at different temperatures: **(a) ***T *= 600°C, **(b) ***T *= 700°C, **(c) ***T *= 800°C. NW shown on **(d) **are grown at *P*_tot _= 3 Torr, and at a ratio *P*_HCl_/*P*_SiH4 _= 4 for 15 min at 800°C. In this condition, there is no tapering of the NW. The inset in Figure 4d shows a SEM image of the catalyst after growth. The cylindrical-faceted shape is typical of VSS growth.

The effect of temperature on the NW growth with a high *P*_HCl_/*P*_SiH4 _ratio (*P*_HCl_/*P*_SiH4 _= 3.3) was investigated. Figure 7 shows NW grown at 600, 700, and 800°C. The NW growth occurs from 700°C and the density of straight NW increases with the temperature, as well as the tapering effect. Another NW growth has been carried out at lower pressure, for a comparable *P*_HCl_/*P*_SiH4 _ratio, but at lower HCl and SiH_4 _partial pressures. As can be seen in Figure 7d, the low total pressure combined with the high *P*_HCl_/*P*_SiH4 _ratio allows avoiding the tapering of the NW and keeping high density and length.

The SEM images of the catalyst (Figure 7d inset) suggest that it remains solid during growth. Indeed, the cylindrical-faceted shape is completely different from the semi-spherical shape typical of Au catalysts after a VLS growth. XRD diffraction measurements performed after the NW growth show that the catalyst particle at the NW tip are hexagonal Pd_2_Si. As expected according to the SEM images, there are no preferential directions for the NW growth.

It has been seen that the use of alternative catalysts such as Pt, Ni, and Pd silicides for the growth of Si NW requires high temperatures. Indeed, the growth occurs through VSS process which consumes much more energy than VLS, mainly because of the diffusion through or at the surface of a solid catalyst. Working at temperatures above 700°C implies an important uncatalyzed growth rate. It has been shown that this uncatalyzed growth can considerably be lowered by using gaseous HCl allowing the growth of less- or non-tapered NW. Moreover, the presence of HCl in the gas phase increases the NW vertical growth rate. This could be explained by an increased probability of silane molecules' decomposition on the catalyst because of an important Cl coverage of the surface. The possibilities of interactions between HCl and catalysts leading to an increase of the NW growth rate are not rejected, but this would require a more thorough study.

## Mechanical characterization

Among the numerous NW's potential applications, electromechanical systems have attracted an increasing interest for the past few years [27]. The manipulation and exploitation of NW for such device requires accurate knowledge of their mechanical properties at the single object level. An AFM multipoint-bending protocol allowing as-grown single NW characterization has been developed by Gordon et al. [32]. It consists in measuring the deflection of an AFM cantilever while performing approach-retract curves at various positions along the length of a cantilevered NW. This approach allows the measurement of single NW's Young modulus and spring constant, and alleviates uncertainties inherent in single point measurement. This AFM-based mechanical testing has been carried out on Si and GaN NW grown with Au catalyst or without catalyst, respectively.

**Figure 8:**
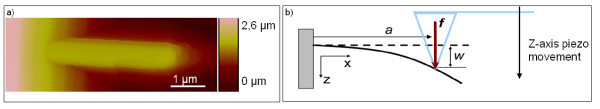
**Single NW mechanical characterization**. **(a) **AFM tapping-mode image of a GaN NW. **(b) **Principle of mechanical measurement on a single NW where *w *is the NW deflection when a force *f *is applied at a position *a*. The cantilever deflection is measured as an indirect measurement of *w*.

Cantilevered NW are imaged in tapping mode and approach-retract cycles are performed at different locations along the NW length (Figure 8). During these cycles, the NW is deformed by the AFM tip, deflection of which is recorded as an indirect measurement of the actual NW deflection. The force-distance curves represent the force applied by the tip (*f*_tip_) as a function of the *z*-axis piezo movements. Owing to theses curves, it is possible to calculate the NW spring constant at each measurement location. The NW Young's modulus can be obtained from the differential equation which describes *w*(*x*), the NW deflection along its length as a function of *f*, and the force applied at *x *= *a*, in the limit of small deflections.(1)

where *E *is the Young's modulus, and *I *= π*r*^4^/4 is the moment of inertia.

A stress-strain relation, where an effective wire spring constant (*k*_wire_) can be defined, is given by solving Equation (1) using appropriate boundary conditions:(2)

Therefore,(3)

With the radius of the NW *r *being deducted from the taping mode scan of the NW, a linear fit of *k*_wire_^-1/3^, as a function of the forcing location *a*, allows the calculation of *E*.

**Figure 9:**
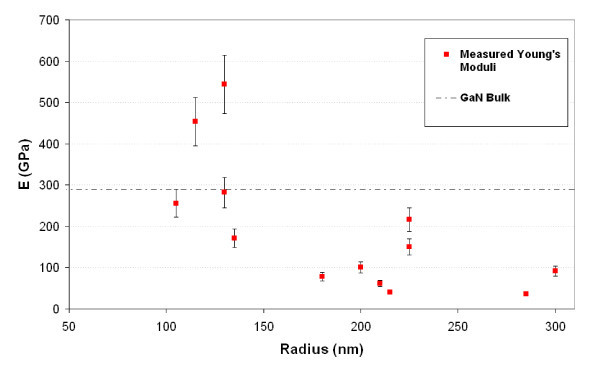
**Young's moduli of GaN NW as a function of the NW radius**. The error bar is estimated according to the following formula: Δ*E*/*E *= 3|Δ*a*/*a*| + 4|Δ*r*/*r*|. The dashed line represents the GaN bulk modulus in the [0001] direction.

Si NW grown along the (111) direction have been tested (results not shown). As expected [32], the measured Young's moduli are comparable to the bulk Si young modulus along the (111) direction. Figure 9 shows the Young modulus of GaN NW with *r *ranging from 100 to 300 nm determined according to this method. GaN NW grow along the *c*-axis ([0001] direction) [33], and the doted line on the graph represents the bulk's Young's modulus along the same direction. As one can see, *E *tends to decrease when the radius increases and becomes much lower than the bulk modulus above *r *= 150 nm. The same behavior has already been reported for GaN [34] and for ZnO NW [35]. A possible explanation could be a diminution of the defect inside the crystal with the diminution of the diameter. As can be seen in ref [33], the section of GaN NW can be irregular from one NW to another which could explain the wide dispersion of the Young's moduli. Moreover, the NW's cross section becomes more and more irregular, and the crystalline quality decreases as the NW diameter increases [33]. This could explain such a decrease of the GaN NW's Young's moduli when the NW diameters increase. This aspect constitutes the main limit of this method; this is why NW with a regular cylindrical diameter are required to obtain reliable results.

## Conclusion

This article reviewed different metal-mediated methods to synthesize Si and SiGe NW. First, gold-assisted synthesis of SiGe NW from 350 to 400°C on Si(111) substrates has been presented. The possibility to obtain a wide range of composition (0 to 50% Ge in SiGe) by varying the gas flow ratio was shown. Second, the growth of silicon NW with silicides catalysts, such as PtSi, NiSi, and Pd_2_Si was reported. Those catalysts present an alternative to gold for the growth of NW with optimized electrical properties. The NW are grown through the VSS process which requires working at high temperatures. The uncatalyzed growth rate, classically important under these conditions, is inhibited by using gaseous HCl. It allows Cl surface coverage that impedes the precursor adsorption and decomposition thus preventing the NW to be tapered. Finally, AFM-based mechanical characterization of single GaN NW is presented. It is shown that the apparent NW's Young's modulus seems to increase when the NW's diameter decreases. This could be explained by a reduction of the defect in small diameter NW and by an irregular cross section of the NW when the diameter increases.

## Abbreviations

CVD: chemical vapor deposition; NW: nanowires; SC: semiconductor; SEM: scanning electron microscopy; TEM: transmission electron microscopy; XRD: X-ray diffraction.

## Competing interests

The authors declare that they have no competing interests.

## Authors' contributions

AP carried out the SiGe NW growth,SEM characterization, analysis and interpretation of the data and drafted the manuscript. TB conceived the study and carried out its coordination,the analysis and interpretation of the data, participated to the growth of NW, and revised the manuscript. FD carried out the growth of Si NW, SEM characterization, analysis and interpretation of the results. PG, FO, participated to the growth of Si and SiGe NW. BS and GR carried out the substrates preparation prior to growths and participated to the SEM characterization of the NW. LLR carried out the TEM analysis, MK carried out the AFM measurements, LM participated to the revision of the manuscript, JK carried out the Raman measurements, HR carried out the XRD measurements. All authors read and approved the final manuscript.
